# 小细胞肺癌诊疗新进展

**DOI:** 10.3779/j.issn.1009-3419.2019.06.05

**Published:** 2019-06-20

**Authors:** 晓霞 崔, 鹏 宋, 力 张

**Affiliations:** 100730 北京，中国医学科学院北京协和医学院北京协和医院呼吸与危重症医学科 Department of Pulmonary and Critical Care Medicine, Peking Union Medical College Hospital, Chinese Academy of Medical Science & Peking Union Medical College, Beijing 100730, China

**Keywords:** 肺肿瘤, 分子生物学, 抗新生血管生成药物, 分子靶向治疗, 免疫检查点抑制剂, Lung neoplasms, Biology, Antiangiogenic agents, Molecular-target therapy, Immune checkpoint inhibitors

## Abstract

小细胞肺癌（small cell lung cancer, SCLC）是一种恶性程度高、病情进展快、预后差、易复发的难治性癌症。在过去的30余年中，SCLC以化疗和放疗为主的传统治疗策略并未出现明显变化，临床需求的有效治疗方式迫在眉睫。精准医学的快速发展揭示了SCLC的分子生物学特征，故其诊疗进入一个新的时代指日可待。目前已有研究显示抗新生血管生成药物、免疫治疗等一定程度上提高了SCLC治疗的疗效，并且有涌现出更多的关于SCLC诊疗方式的研究，从而开辟了SCLC治疗的新领域，给患者带来更多生存获益。针对SCLC分子病理的靶向治疗、抗新生血管生成药物、免疫治疗方面新的研究崭露头角，本文对SCLC新的诊疗方式进行综述，以示读者，为其临床治疗提供新的指导。

## Abstract (en)

Small cell lung cancer (SCLC) is a refractory cancer with high degree of malignancy, rapid disease progression, poor prognosis and easy recurrence. In the past 30 years, the traditional treatment of SCLC, mainly chemotherapy and radiotherapy, has not changed significantly, and the effective treatment method for clinical needs is extremely urgent. The rapid development of precision medicine has revealed the molecular biological characteristics of SCLC, so its diagnosis and treatment will into a new era. At present, some studies have shown that anti-angiogenic drugs, immunotherapy and so on have improved the efficacy of SCLC treatment to some extent, and there are more studies on the diagnosis and treatment of SCLC, so a new field of SCLC treatment are coming and bringing more survival benefits to patients. New studies on targeted therapy, anti-angiogenesis drugs and immunotherapy of molecular pathology of SCLC are emerging. This paper reviews the new diagnosis and treatment methods of SCLC to provide new guidance for its clinical treatment.

目前世界范围内发病率和死亡率最高的恶性肿瘤是肺癌，全球年新发肺癌患者约209.3万人，死亡176.1万人，而中国年肺癌新发及死亡人数则分别为约78.1万和62.6万^[1]^。中国癌症死亡的首要元凶是肺癌^[2-5]^。小细胞肺癌（small cell lung cancer, SCLC）是一种起源于支气管黏膜上皮的Kulchitsky细胞的异源性神经内分泌肿瘤，是肺癌中侵袭性最强的亚型，约占肺癌的15%-20%^[6, 7]^。98%的SCLC归因于吸烟，其特点是恶性程度高、侵袭性很高、病情进展快、早期出现远处转移等^[8, 9]^。

2015年，世界卫生组织（World Health Organization, WHO）重新将肺部肿瘤的组织学进行了分类界定，将SCLC归类为神经内分泌肿瘤^[10]^。SCLC的疾病分期一直沿袭1973年美国退伍军人管理局肺癌研究组（Veterans Lung Study Group, VALG）提出的2分期系统，即局限期（limited-stage SCLC, LS-SCLC）和广泛期（extensive-stage SCLC, ES-SCLC）^[11]^。在临床实践中，鉴于VALG法的实用性很强，美国国立综合癌症网络（National Comprehensive Cancer Network, NCCN）指南建议SCLC分期采取美国癌症联合委员会（American Joint Committee on Cancer, AJCC）的国际恶性肿瘤标记符号（Tumor Node Metastasis, TNM）（第7版）分期方法与VALG法相结合^[12, 13]^，即LS-SCLC是指可被单个放射野包括覆盖的肿瘤组织，包括TNM分期的Ⅰ期-Ⅲ期（任意T、任意N、M0），除外多发肺结节的T3-4或肿瘤过大不能耐受放疗的患者；ES-SCLC是指超出单个放射野范围的任意肿瘤，TNM分期的Ⅳ期（任意T、任意N、M1a/b）和多发肺结节或肿瘤过大不能耐受放疗的T3-4患者^[14-16]^。

2019年SEER数据库公布了1973年-2010年肺癌5年的生存率，研究发现4种不同病理类型的肺癌的5年生存率都呈增加的趋势，SCLC的改善却甚微，仅为2.8%-7.2%^[17]^。2018年中国临床肿瘤协会（Chinese Society of Clinical Oncology, CSCO）制定的SCLC临床指南推荐T1-2N0患者可考虑手术切除，术后N1-2患者接受辅助放疗，术后辅助放疗同步或序贯均可^[18-20]^。

几乎2/3的SCLC确诊时处于晚期，失去手术或根治的机会，化疗依然是标准疗法^[21-23]^。自1980年以来，依托泊苷联合铂类，结合胸部放疗是SCLC的一线金标准治疗方案^[24-27]^。虽然初期SCLC患者对放化疗特别敏感，但是大部分患者会在2年内复发或转移而死亡^[28-30]^。目前临床应用中二线化疗方案为拓扑替康药物治疗^[31-34]^。2017年世界肺癌大会（World Conference on Lung Cancer, WCLC）报道了一项基于美国、欧洲、日本人群的ES-SCLC治疗模式的真实世界临床研究，94%的ES-SCLC一线治疗选择含铂化疗方案，顺铂+伊立替康方案在日本更为广泛使用。平均19%的ES-SCLC接受了二线治疗，在铂类耐药的患者中，二线依然接受含铂化疗方案的比例分别为：美国27%、欧洲11%、日本36%。即使顺铂敏感的患者，美国及欧洲二线治疗方案中选拓扑替康的更为常见。此外，该真实世界研究提示当前SCLC的二线治疗模式缺乏，疗效差^[35]^。综上，SCLC一线复发率高，二线方案有限且疗效极差，三四线之后几乎无公认的治疗方案，因此改善SCLC的治疗一直是临床棘手的问题，亟待探索新的药物。

目前已有研究显示抗新生血管生成药物、免疫治疗等一定程度上提高了SCLC治疗的疗效^[36]^，并且有涌现出更多的关于SCLC诊疗方式的研究，从而开辟了SCLC治疗的新领域，给患者带来更多生存获益。针对SCLC分子病理的靶向治疗、抗新生血管生成药物、免疫治疗方面新的研究崭露头角，本文对SCLC新的诊疗方式进行综述，以示读者，为其临床治疗提供新的指导。

## 1 SCLC的分子生物学研究新进展

### 1.1 SCLC的分子生物学特征

SCLC与普通型肺癌相比，不仅在组织形态上具有明显的异质性，而且在肿瘤分子生物学行为以及对药物治疗的反应性上也存在着显著差异。研究表明，SCLC存在极度的免疫抑制和多种免疫细胞浸润，如CD45^+^ T细胞、CD8^+^、CD4^+^、肿瘤相关的巨噬细胞（tumor associated macrophage, TAM）、IL-2、调节性T细胞（regulatory T cell, Treg）等细胞，并且与其预后存在相关性，免疫逃逸和抑制在SCLC的发生和发展中扮演着重要作用^[37-42]^。Treg细胞可分泌抑制性细胞因子，包括IL-10、IL-35等，动物实验证实清除肿瘤微环境中的Treg细胞可增强抗肿瘤免疫^[39-41]^。TAM有M1、M2两种表型，分别发挥抗肿瘤和促肿瘤活性。肺癌动物实验显示TAM数量减少可导致肿瘤体积缩小^[41]^。2017美国癌症研究协会年会（American Association for Cancer Research, AACR）的一项回顾性研究共纳入了13, 020例患者，包含6个瘤种、23项有关Nivolumab的临床研究，显示：SCLC患者的程序性死亡受体1（programmed death protein ligand 1, PD-L1）≥1%的患者占15.6%；肿瘤表面PD-L1表达约为SCLC的10%，且无预测作用；免疫细胞PD-L1表达更高，接近40%^[43]^。最新研究表明，SCLC与极高的突变率有关，普遍出现*RASSF1*（> 90%）、*FHIT*（80%）、*TP53*（75%-90%）、*RB1*（60%-90%）、*RARB*（72%）、*FUS1*（40%-60%）基因突变^[44, 45]^。此外，还观察到*CDKN2A*、*SLIT2*、*EPHA7*、*PTEN*、*PIK3CA*、*EGFR*、*KRAS*等基因突变^[46]^。其次，SCLC中MYC家族成员MYC-L1 > N-MYC > C-MYC的扩增普遍存在（15%-30%）^[47]^。另外SCLC还存在其他基因变异，如：*FGFR1*扩增（6%）、*SOX2*扩增（27%）、染色体修饰中的复发点突变（*CREBBP*、*EP300*、*MLL*）（10%-20%）、*NOTCH*家族基因失活突变（25%）和*FMN2*、*NEZH2*肿瘤获得性耐药的染色体重组相关调节因子^[48, 49]^。

### 1.2 SCLC的NOTCH通路研究进展

跨膜受体蛋白NOTCH通路在胚胎发育中起核心作用，干细胞和祖细胞调节，即人类肿瘤中起作用，包括SCLC，不在成人健康组织中表达^[50]^。Notch失活突变可以诱导非神经内分泌肿瘤细胞或肿瘤前体细胞神经内分泌分化，继发性SCLC起源假说表明Notch-ASCLl-RB-p53信号通路通过Notch失活突变驱动并维持SCLC表型^[51]^。Notch信号通路的活性在决定细胞命运（包括增殖、分化以及凋亡）过程中既可以抑制肿瘤也可以致癌。在肺发育过程中，Notch通路激活可抑制前体细胞向神经内分泌分化。在大部分SCLC中，Notch基因突变的功能丧失以及异位Notch激活表现出肿瘤抑制作用。最新研究发现^[52]^，10%-50%小鼠肺癌模型或人类肿瘤细胞发生Notch途径内源性激活导致发生神经内分泌向非神经内分泌转化，这种转化由转录抑制因子Nrsf介导，抑制神经内分泌基因表达。非神经内分泌Notch激活SCLC细胞生长缓慢，但对化疗不敏感并且向内分泌肿瘤细胞提供营养支持，表现出致癌作用。更为关键的是，临床前模型发现Notch可阻断化疗对肿瘤生长的抑制和复发延缓作用。研究发现，部分SCLC通过Notch信号通路激活生成独特的微环境，Notch信号可以作为SCLC患者预计化疗效果的生物标志物。非典型NOTCH配体诱导关键神经内分泌转录因子ASCL1，ASCL1的表达与LSD1抑制剂对SCLC的敏感性相关，ASCL1高表达的SCLC移植瘤接受LSD1抑制剂能够显著抑制肿瘤生长，延长生存；ASCL1不表达的SCLC移植瘤接受LSD1抑制剂治疗不能抑制肿瘤生存，不能改善生存^[50-53]^。LSD1抑制剂通过调节NOTCH-ASCL1轴，抑制剂SCLC的增殖和生长，ASCL1表达可能是LSD1抑制剂治疗潜在的预测标志物^[53]^。组蛋白赖氨酸特异性去甲基化酶1（LSD1），协同组蛋白甲基化和乙酰化，控制靶基因转录，是多种发挥抑制功能的复合物的组成成分。高通量筛选发现LSD1抑制剂能抑制部分SCLC细胞系增殖，信号通路分析发现，LSD1抑制剂治疗后对SCLC细胞系相关基因的影响主要集中在Notch信号和神经分化相关的基因。在LSD1抑制剂敏感的SCLC细胞中，LSD1治疗使NOTCH信号活化，基因表达上调，Notch信号的致癌基因*ASCL1*下调^[53]^。Delta-like ligand 3（DLL3）蛋白抗体是Notch信号的配体之一，是Notch信号的抑制因子，可直接影响Notch下游的靶基因ASCL1，DLL3过表达与神经内分泌肿瘤的发生相关，SCLC的DLL3高表达占85%。在多数SCLC中ASCL1异常表达，促进DLL3表达，DLL3与Notch1受体结合，抑制Notch信号活化。HES1、HEY1抑制ASCL1表达，SCLC中Notch通路失活，靶基因*HES1*、*HEY1*的表达下调，对ASCL1抑制剂解除，促进SCLC的发生发展^[50]^。

### 1.3 SCLC的血管内皮生长因子研究进展

血管内皮生长因子（vascular endothelial growth factor, VEGF）与其受体（vascular endothelial growth factor receptor, VEGFR）结合后促进血管生成，为肿瘤细胞供氧，并促进免疫抑制环境形成^[52]^。抗血管生成靶向药物可抑制肿瘤组织生长，但不能消除肿瘤细胞，因此其单药不能发挥持久的抗瘤活性。研究发现VEGF/VEGFR通路与免疫系统存在相互促进的作用^[54]^，为两者的联合治疗提供了理论基础。VEGF家族由VEGF-A、VEGF-B、VEGF-C、VEGF-D和VEGF-E以及它们的三个VEGF受体（VEGFR1-3）所组成，VEGF信号通路可使内皮细胞的增殖、迁移和侵袭性增强，从而介导肿瘤的血管生成，SCLC患者的VEGF水平较高，且与肿瘤分期、疾病进展、化疗耐药以及不良预后有关^[55]^。

### 1.4 SCLC的DNA损伤修复通路研究进展

目前研究发现，SCLC的发生还与DNA损伤修复通路的部分基因异常表达有关，如聚二磷酸腺苷核糖多聚酶（PARP）、*MGMT*基因的沉默等。PARP家族与DNA损伤修复有关，通过其最重要的剪切修复作用（base excision repair, BER）或者包括HRR与NHEJ在内的修复信号通路发挥作用^[56]^。SCLC的PARP高表达可能与耐药和肿瘤细胞基因毒性应激抵抗相关^[57]^。基因敲除或药物抑制PARP酶可以增强DNA损伤类化疗药物与粒子放疗的细胞学毒性^[58, 59]^，使得化疗、粒子放疗引起的细胞毒性得以巩固，不易修复。

## 2 针对SCLC生物学特征治疗的初步结果

### 2.1 Rova-T靶向治疗

ROVA-T是抗DLL3蛋白抗体Rovalpituzumab与细胞毒素Tesirine的偶联药物链接细胞表面上的不典型NOTCH配体DLL3，并且传导DNA损伤药物双重毒性^[58]^。免疫组织化学检测显示约80%的SCLC肿瘤组织和肿瘤细胞表面存在DLL3表达，因此DLL3可能是SCLC理想的治疗靶点。ROVA-T是SCLC第一个靶向治疗药物，利用表达在肿瘤细胞表面的DLL3识别肿瘤细胞并将细胞毒性药物输送到肿瘤细胞内，达到定向杀死肿瘤细胞的作用^[59]^。与传统标准方案相比，Rova-T单药在复发/难治性SCLC，二线和三线治疗上取得了较好的疗效，可以提高疗效，延长患者总生存，毒副作用可管理。2018年美国临床肿瘤学会（American Society of Clinical Oncology, ASCO）报道的一项Ⅱ期临床试验TRINITY研究（NCT02674568）共纳入199例经治患者，三线及之后接受ROVA-T 0.3 mg/kg，d1，每6周1次，共2次的治疗。研究显示DLL3高表达的晚期SCLC患者中位无进展生存期（progression-free survival, PFS）为4.1个月，总生存期（overall survival, OS）为6.7个月^[60]^。

### 2.2 PARP抑制剂

PARP通过抑制肿瘤细胞DNA损伤修复、促进肿瘤细胞发生凋亡，从而增强放疗以及烷化剂和铂类药物化疗的疗效^[61-63]^。PARP抑制剂在SCLC的研究仍在探索阶段，以1期/2期研究，联合治疗为主，涵盖一线、二线和维持治疗。奥拉帕尼（olaparib）是目前在研的一种口服的选择性PARP抑制剂，主要针对PARP1/2靶点，可选择性的抑制DNA的修复^[64]^。2018年ASCO报道的olaparib联合替莫唑胺治疗晚期SCLC的单臂Ⅰ期/Ⅱ期临床试验入组了30例之前接受过≥1种铂类化疗治疗后进展的SCLC患者接受olaparib联合替莫唑胺的梯度剂量治疗。其结果显示：Ⅰ期/Ⅱ期客观缓解率（objective response rate, ORR）为41.4%。olaparib联合替莫唑胺的Ⅱ期研究的中位无进展生存期PFS为2.8个月，中位OS为7.3个月^[65]^。

Veliparib是目前在研的另一类口服PARP酶抑制剂，临床前研究（细胞系和动物模型）显示该药物可增强含铂双药化疗疗效。一项Veliparib联合CE双药一线治疗ES-SCLC的研究提示，Veliparib联合CE双药一线治疗ES-SCLC临床获益，联合Veliparib增加了血液学毒性，但不影响化疗药物剂量^[66]^。目前，Veliparib在SCLC的研究集中在一线治疗，主要研究方向是联合化疗，但是不管是初治SCLC还是复发SCLC与化疗相比，均无PFS和OS获益。

### 2.3 血管靶向治疗

我国自主原创新药盐酸安罗替尼作为新型多靶点小分子TKI药物，主要作用于VEGFR、PDGFR、FGFR以及c-Kit等^[67]^。ALTER1202研究是一项安罗替尼对照安慰剂三线及三线以上治疗SCLC的随机、双盲、安慰剂对照、多中心Ⅱ期临床试验（ALTER1202, NCT03059797）^[68]^。该试验纳入120例患者，主要为ES-SCLC患者，2:1随机分为安罗替尼组（*n*=82）和安慰剂组（*n*=38）。结果显示，25%的患者入组前就存在脑转移，三线治疗的比例为75%。与安慰剂对比，安罗替尼将主要终点PFS延长了3.4个月（4.1个月*vs* 0.7个月），将疾病进展风险降低了81%（*P* < 0.000, 1），疾病控制率从13%提高到71%，这可能与安罗替尼作用于多个靶点密不可分。该研究另一亮点是PFS的亚组分析中，所有亚组接受安罗替尼治疗均有显著获益，尤其是对于脑转移和三线治疗的患者获益更为显著，脑转移患者的PFS延长了3个月[3.8个月 *vs* 0.8个月，风险比（HR）为0.15，*P*=0.003]，三线治疗的患者PFS延长了3.4个月（4.1个月 *vs* 0.7个月，HR为0.15，*P* < 0.001）。虽然安罗替尼与安慰剂比较，ORR没有显著差异（4.9% *vs* 2.6%），但却有很好的疾病控制率，DCR分别为71.6%和13.2%，*P* < 0.000, 1。数据截止到2018年6月30日，OS尚未成熟（成熟度为44.5%），但就目前结果来看，安罗替尼组的OS显著延长了2.4个月，死亡风险下降47%（*P*=0.021）。在ALTER1202研究中，安罗替尼与安慰剂的总体治疗相关不良事件（adverse event, AE）发生率相似，分别为87.7%和74.4%，最常见的治疗相关性AE为高血压、厌食、乏力、手足综合征、促甲状腺激素（thyroid stimulating hormone, TSH）升高、白细胞减少、丙氨酸氨基转移酶（alanine aminotransferase, ALT）升高等。总体来看，该研究中安罗替尼的不良事件与其他瘤种的研究相似，未发生非预期不良事件，耐受性较好。虽然安罗替尼3级-5级AE发生率高于安慰剂（35.8% *vs* 15.4%），但临床易于管理。在安罗替尼组，有4例患者因AE剂量调整，6例因AE停药，只有1例患者死亡可能与药物有关。

基于以上研究结果，可以考虑在后续研究中进一步评估安罗替尼联合化疗在ES-SCLC二线治疗的临床疗效。此外，安罗替尼与PD-1/PD-L1等免疫检查点抑制剂联合是否可以提升疗效，需要进一步探索。

## 3 SCLC的免疫治疗新进展

2012年SCLC开启了免疫治疗的篇章，试验药物逐渐丰富，临床研究逐年增加，治疗方式逐渐多样，为SCLC的治疗带来了新希望^[69]^。抗PD-L1的单克隆抗体（抑制剂）可阻断PD-L1与程序性死亡蛋白-1（programmed death protein-1, PD-1）、CD80的相互作用，从而恢复T细胞功能^[70, 71]^。PD-L1表达是一种适应性反应，能帮助肿瘤逃避免疫系统的检测和清除。PD-L1蛋白表达通常由适应性免疫反应相关的炎症性信号（如IFNγ）诱导，并存在于肿瘤细胞和肿瘤浸润性免疫细胞中。PD-L1与活化T细胞表面的PD-1结合后可向T细胞释放抑制信号，从而阻止T细胞消灭目标肿瘤细胞，保护肿瘤不被免疫系统消除^[72, 73]^。免疫检查点抑制剂联合化疗存在协同作用可能增强抗肿瘤疗效。

最新的研究KEYNOTE-028研究显示单药治疗PD-L1阳性广泛期SCLC，客观缓解率33%，中位总生存时间9.7个月^[74]^。KEYNOTE-158研究显示Pembrolizumab治疗复发性SCLC的疗效依赖于PD-L1表达^[75]^。

Nivolumab可以抑制激活T细胞表面PD-1的表达，增加效应T细胞数量从而增强抗肿瘤效应。CheckMate-032研究了铂类化疗后疾病进展的SCLC患者对Nivolumab的疗效，110例铂类化疗后进展的患者接受了Nivolumab治疗。12%的患者（*n*=13/109; 95%CI: 6.5-19.5）对治疗有反应，未发现与PD-L1表达状态相关；12例部分缓解（11%），1例完全缓解（0.9%）；DOR中位数为17.9个月（95%CI: 7.9-42.1；范围：3.0个月-42.1个月）；10%的患者因不良反应而停用Nivolumab，25%的患者因不良反应停用一次；45%的患者出现严重不良反应^[76, 77]^。Nivolumab+Ipilimumab *vs* Nivolumab单药，ORR和OS更好。基于该研究结果，NCCN指南推荐nivolumab±ipilimumab用于一线治疗6个月内复发的SCLC^[7]^。

IMpower133（Atezolizumab联合顺铂/依托泊苷一线治疗ES-SCLC的全球多中心随机双盲Ⅲ期研究）旨在评估Atezolizumab作为SCLC一线治疗联合卡铂和依托泊苷的有效性和安全性。该研究共纳入403例未接受治疗的ES-SCLC患者，1:1随机接受Atezolizumab联合卡铂和依托泊苷或安慰剂联合卡铂和依托泊苷^[78]^。其结果显示Atezolizumab组的中位OS为12.3个月（95%CI: 10.8-15.9），安慰剂组为10.3个月（*P*=0.006, 9），死亡风险降低了30%。Atezolizumab组的中位PFS为5.2个月（95%CI: 4.4-5.6），而安慰剂组为4.3个月（95%CI: 4.2-4.5）（HR=0.77; 95%CI: 0.62-0.96; *P*=0.017）。与安慰剂相比，Atezolizumab与较高的6个月PFS率（30.9% *vs* 22.4%）相关，12个月PFS率（12.6% *vs* 5.4%）增加1倍以上。两组之间的ORR（分别为60.2%和64.4%）和中位反应持续时间（4.2个月*vs* 3.9个月）没有显著差异^[79]^。IMpower133是近20年来第一项在ES-SCLC一线治疗中显示出总体生存改善的有临床意义的研究。基于该研究，NCCN指南推荐免疫检查点抑制剂用于SCLC的一线治疗^[7]^。

SCLC免疫治疗崭露头角，Nivolumab单药已经被批准用于复发性SCLC三线治疗，Nivolumab联合Ipilimumab或Pembrolizumab单药治疗SCLC是目前得到NCCN指南推荐的二线治疗方案，IMpower133研究是目前唯一SCLC一线治疗得到阳性结果的Ⅲ期研究，被NCCN指南推荐为一线治疗方案^[7]^。免疫治疗能够突破SCLC治疗的困境，需要对耐药的机制、优势人群的筛选、联合治疗的最佳时机和模式以及新的靶点做更多的研究探索。

## 4 总结

综上，SCLC是一种顽固不化、疾病特征不同于其他类型肺癌的肿瘤，恶性程度高，5年生存率低，其治疗手段匮乏，预后不佳。目前研究证实，Rova-T靶向治疗、PARP抑制剂、血管靶向治疗、免疫治疗等对于SCLC均有疗效。但是，面对不同肿瘤负荷、不同生物学特征、不同免疫状态的SCLC患者如何把这些不同的治疗方法精准结合起来制定一个最合理的策略，使其发挥最佳治疗效果，将短期疗效转变为长期疗效，是一个巨大的挑战，是未来SCLC发展方向的重中之重。

由于SCLC接受手术治疗的患者非常少，基因检测方面的研究也相对较少，具体的耐药机理尚不清楚。对于发生化疗耐药的患者，是否可以通过再次活检的方式从基因层面来明确化疗耐药机理，还有待于进一步研究证实。真实世界临床实践亟待新药物，免疫治疗正在探索中，为SCLC带来新的希望，并改变着临床实践，但在维持治疗探索中道路曲折。新型靶向药物或能为SCLC也带来新希望，但需要筛选优势人群。今后的研究中，或许只有深入了解SCLC免疫机制和其相关的免疫微环境，才能将放化疗、免疫治疗、靶向治疗等结合起来，从而开启SCLC诊疗新篇章。
